# CD8^+^ T Cell-Mediated Immunity during *Trypanosoma cruzi *Infection: A Path for Vaccine Development?

**DOI:** 10.1155/2014/243786

**Published:** 2014-07-01

**Authors:** Fernando dos Santos Virgilio, Camila Pontes, Mariana Ribeiro Dominguez, Jonatan Ersching, Mauricio Martins Rodrigues, José Ronnie Vasconcelos

**Affiliations:** ^1^Centro de Terapia Celular e Molecular (CTCMol), UNIFESP-Escola Paulista de Medicina, Rua Mirassol 207, São Paulo 04044-010, SP, Brazil; ^2^Departmento de Microbiologia, Imunologia e Parasitologia, Universidade Federal de São Paulo-Escola Paulista de Medicina, Rua Mirassol 207, São Paulo 04044-010, SP, Brazil; ^3^Departamento de Biociências, Instituto de Saúde e Sociedade, UNIFESP, Campus Baixada Santista, Santos 11015-020, SP, Brazil

## Abstract

MHC-restricted CD8^+^ T cells are important during infection with the intracellular protozoan parasite *Trypanosoma cruzi*, the causative agent of Chagas disease. Experimental studies performed in the past 25 years have elucidated a number of features related to the immune response mediated by these T cells, which are important for establishing the parasite/host equilibrium leading to chronic infection. CD8^+^ T cells are specific for highly immunodominant antigens expressed by members of the *trans*-sialidase family. After infection, their activation is delayed, and the cells display a high proliferative activity associated with high apoptotic rates. Although they participate in parasite control and elimination, they are unable to clear the infection due to their low fitness, allowing the parasite to establish the chronic phase when these cells then play an active role in the induction of heart immunopathology. Vaccination with a number of subunit recombinant vaccines aimed at eliciting specific CD8^+^ T cells can reverse this path, thereby generating a productive immune response that will lead to the control of infection, reduction of symptoms, and reduction of disease transmission. Due to these attributes, activation of CD8^+^ T lymphocytes may constitute a path for the development of a veterinarian or human vaccine.

## 1. Relevance of Chagas Disease

*Trypanosoma cruzi* is the etiologic agent of Chagas disease, also known as American trypanosomiasis. Infection with this protozoan parasite currently affects approximately 10 million people (WHO 2013) and is the major cause of nonischemic heart disease worldwide. In Latin America, Chagas disease causes an annual loss of 750,000 years of productivity in addition to a loss of 1.2 billion dollars. Despite the fact that most infected individuals are present in endemic areas of Latin America, an increasing number of cases have been reported outside these areas in recent decades, mainly due to the migratory flux of infected people to other regions. Thus, it is estimated that there are approximately 300,000 currently infected people in the United States, 5,500 in Canada, 80,000 in Europe, 1,500 in Australia, and 3,000 in Japan.

Furthermore, there is no effective chemotherapeutic treatment against this disease. Drugs conventionally used for the treatment of infected individuals were developed in the 1960s (nifurtimox and benznidazole). Being outdated, these drugs are highly toxic, have variable efficacy and weak activity in immunocompromised patients, and require long treatments, and the cost of treatment is high, starting from USD 1,000 per year per patient.

Currently, vectorial control using insecticides is the main method to prevent the transmission. Although vectorial transmission has been controlled in some Southern Cone countries [1] there are reports of insect vector resistance to insecticides in other regions, such as the Gran Chaco [2]. Still, some insect vector species are particularly difficult to control by insecticides; thus, it is likely that control by insecticides will only keep transmission at low levels without eradicating the disease [2].

It should be also noted that vector control does not block transmission by secondary routes, which are the main modes of transmission outside of endemic areas or where the insect vector has been controlled or is absent. The secondary routes are (i) mother to child transmission and blood transfusion and (ii) organ transplantation from infected people. In order to reduce infections by blood or organ transplants, it is critical to analyze donated blood and organs for the presence of parasites [3].

Considering the number of affected people, the economic impact in endemic areas, the recent dispersion of infected people, the inefficiency and cost of treatment, and the lack of effective methods to control transmission, the difficult task of developing a vaccine against infection by* Trypanosoma cruzi* has been resurrected.

## 2. Relevance of the Development of a Vaccine for Chagas Disease

In a recent study, Lee et al. generated a computer model to simulate the economic impact of a prophylactic vaccine for Chagas disease in Latin America [4]. This study demonstrated that a vaccine would be beneficial even if it had low protective efficacy (25%), even in places where the risk of becoming infected is less than 1%, and even if the vaccine cost is USD 20.00. A vaccine costing more than USD 200.00 would only be economically advantageous if it had efficacy greater than 50% and was used in places where the risk of becoming infected was greater than 20%. Based on these studies, several groups have been focusing on the development of a vaccine against Chagas disease.

## 3. Clinical Data of Chagas Disease

Chagas disease has two distinct clinical phases: acute and chronic. In the acute phase, individuals exhibit patent parasitemia that begins one to two weeks after infection. In this phase, the infection has variable symptoms from asymptomatic to intensely symptomatic and includes risk of death. Except Romaña sign that is characterized as a swelling at the site of infection, signs and symptoms related to the acute phase of Chagas disease symptoms are most of the times nonspecific and consequently it is difficult to detect the infection in the initial stage. In the acute phase, there is strong activation of the immune system with high levels of plasma cytokines, intense activation of B and T lymphocytes, and inflammatory reactions in the infected tissues (WHO 2013).

In most cases, the acute phase parasitemia is controlled gradually, presumably due to the host adaptive immune responses, and it is reduced to subpatent levels. After controlling acute parasitemia, the chronic phase is initiated. In this phase, it is possible to detect specific antibodies by different serological tests and, in many patients, the parasites can be isolated by blood culture or by xenodiagnosis. These tests are routinely used for parasitological exams (WHO 2013). The chronic phase can last for the entire life of the patient and is initially asymptomatic. After years of initial contact with the parasite, 40% of infected persons develop symptomatology, while the remaining 60% remain asymptomatic throughout life.

In approximately 30% of cases, patients develop cardiac symptoms (associated with severe myocarditis), and in the remaining 10%, there is damage to the digestive system with infiltration of lymphocytes and neuronal degeneration, leading to the development of megacolon or megaesophagus [5]. Despite the fact that the causes of the immunopathology are not precisely known, several evidences indicate that parasite persistence is a critical factor for the development of Chagas disease [5, 6]. Accordingly, extensive destruction and fibrosis of the heart would be a result of the inflammatory reaction required to eliminate the parasites in the tissues [7]. Nevertheless, whether the parasite has to be present within the heart tissue is still a debatable question. Although most studies have found parasites in this tissue by using detection of DNA by PCR, live parasite has not been detected [8].

## 4. Immune Response During* T. cruzi* Infection

### 4.1. Patients with Chagas Disease

In order to establish a successful chronic infection, there must be a balance between parasite multiplication in the tissues and host immune control that will keep the host alive. In this regard, the host immune response is part of the parasite life cycle. Without proper control of the immune response, the host may die, and the parasite life cycle is broken.

During human infection, protective antibodies are mainly directed towards the Gal epitope containing the *α*Gal *α*1,3-Gal *β*1,4-GlcNAc, which is present in the majority of mucin glycoproteins on the surface of* T. cruzi* trypomastigotes [9]. These antibodies are present in high concentrations during the patent infection and the entire chronic phase and are markers for active human infection [10]. These antibodies kill the parasite independently of the presence of complement or cells, and their activity is facilitated by the presence of specific antibodies for the catalytic domain of the TS enzyme that inhibit sialic acid addition to the surface of trypomastigotes [11]. Whether antibodies directed to other parasite targets are also important in immunity against infection remains to be determined.

Specific T lymphocytes against* T. cruzi* antigens can be detected in most individuals during chronic infection [12]. There is no direct evidence that these cells are important for host resistance. However, it is possible that they exert antiparasitic activities similar to those observed during experimental infection (see below). Events leading to the depression of the immune response, such as acquired immunodeficiency or immunosuppressive treatments (e.g., AIDS), cause relatively rapid onset of symptomatic forms of the disease in some patients with Chagas disease [13]. In some cases, there is reappearance of patent parasitemia, that is, characteristic of the acute stage of infection [14]. Therefore, it seems clear that the adaptive immune response maintains the low levels of parasitemia observed in patients with Chagas disease during the chronic phase.

Comparative studies of the cellular immune responses in patients who develop clinical symptoms, such as chronic chagasic cardiomyopathy (CCC), have led to conflicting results. Some researchers have associated the high production of IFN-*γ* with CCC [15, 16]. Based on this, polymorphisms in the promoter gene regions that encode BAT-1 and IL-10 anti-inflammatory proteins suggest that production of IFN-*γ* is more frequent in patients with CCC [17, 18]. Additionally, Tregs have been correlated with clinical CCC improvement [19]. Thus, patients able to control the inflammatory response in the myocardium may have less tissue damage and milder disease. In contrast, other groups have suggested the opposite: higher production of proinflammatory cytokines, such as IFN-*γ* and IL-17, correlates with an improvement in the clinical picture of chronic Chagas disease [20, 21]. In these cases, CCC would develop due to increased expression of molecules that inhibit the activation of specific T lymphocytes, leading to exhaustion of the specific immune response [21, 22].

### 4.2. Experimental Murine Models

In an attempt to identify the molecules and mechanisms associated with resistance to* T. cruzi* infection, immunologists have employed a number of genetically modified mice. It has been described that resistance to* T. cruzi* infection requires activation of the innate and adaptive immune responses directed at different stages of parasite development. In the case of the innate immune response, several mechanisms operate in order to partially control multiplication of the parasite. Accordingly, mice that are genetically deficient in Toll-like receptors (TLRs) and their adaptors (MyD88 and TRIF), the NOD-like receptors (NLR), or inflammasome activators are more susceptible to experimental* T. cruzi* infection [5, 23, 24]. These molecules provide signals for the secretion of mediators, such as TNF-*α*, IFN-*γ*, and IL-12, which in turn are critical for resistance against experimental infection [25]. Upon activation of the innate immune response, there is intense activation of CD4^+^ and CD8^+^ T lymphocytes, as well as B cells. Likewise, in innate immunity, mice genetically deficient in CD4 or CD8 are also extremely susceptible to infection [26]. These T lymphocytes act by producing type 1 cytokines, such as IFN-*γ*. However, in the case of CD8^+^ T lymphocytes, direct cytotoxicity mediated by perforin may be important for resistance against infection with some strains of* T. cruzi* [27–29]. Finally, in addition to classical innate and adaptive immune responses, NK cells are also activated and produce IFN-*γ* [30].

## 5. Role of CD8^+^ T Cells during* T. cruzi* Infection

### 5.1. Patient with Chagas Disease

In the cardiac lesions of chronically infected individuals, the inflammatory infiltrate is largely dominated by CD8^+^ T cells with a minor proportion of CD4^+^ T cells, B cells, plasma cells, macrophages, eosinophils, and mast cells [22]. This predominance suggests an important role for CD8^+^ T cells during infection [31–33]. Given the difficulty of obtaining cardiac tissue from chagasic individuals, analysis of the specificity of intracardiac CD8^+^ T lymphocytes from patients with Chagas cardiomyopathy is limited to a single study involving a small group of patients with HLA-A0201 [34]. In this study, it was shown that patients had specific CD8^+^ T lymphocytes to some of the 26 peptides tested from FL-160 or cruzipain antigens in the peripheral blood. In the same study, cells specific for one of the cruzipain peptides were isolated from the cardiac tissue of a patient. Cells with this same specificity were also present in the peripheral circulation [34].

The frequency of specific CD8^+^ T lymphocytes was lower in the peripheral blood of chagasic patients with severe disease than in asymptomatic or mildly symptomatic patients. Furthermore, the stage of differentiation of these T cells was different between these two groups. Patients with severe disease had fewer early memory cells (CD45RA^−^, CD27^+^, and CD28^+^) and a higher number of late memory cells (CD45RA^−^, CD27^−^, and CD28^−^). These results suggest that protection against this disease may be associated with an increased frequency of highly competent CD8^+^ T lymphocytes in the early state of differentiation [35]. Infected people in the chronic phase without cardiac symptoms or with mild symptoms had a higher frequency of specific CD8^+^ T lymphocytes producing IFN-*γ* than people who developed severe symptoms. Therefore, the severity of the disease may be related to the specificity and quality of the CD8^+^ T cell response generated during infection [20]. In addition, because it is a chronic infection and the antigen persists, it is possible that the cellular response is driven to exhaustion [35]. However, this is not the case of the mouse model. One possible explanation is that the murine model has a very short life span when compared to humans, and then, mice do not develop exhausted T cells.

The specificity of the CD8^+^ T lymphocyte response was analyzed, and several TS epitopes restricted to HLA-A0201 were identified. Due to the high degree of polymorphism of the human HLA plus the divergent specific response among different strains of* T. cruzi*, it is difficult to establish a correlation between the human immune response and resistance to infection [36]. To identify the target specificity of the CD8^+^ T lymphocyte response from patients in the chronic phase, Alvarez et al. (2008) carried out extensive screening of TS-derived peptides predicted to bind common HLA molecules. The most prevalent HLAs in the population were divided into six supertypes comprising one or more HLA alleles present in 95% of the human population irrespective of ethnic origin. The results of this study indicated that promiscuous TS epitopes, which were able to bind to HLA-A02, HLA-A03, and HLA-A24, are the main targets of memory CD8^+^ T lymphocytes in chronic chagasic patients. Responses to these epitopes were detected in 13 of 25 asymptomatic individuals. This approach suggests that a specific set of HLAs restricted to these peptides can be used to measure the functional activity and frequency of CD8^+^ T lymphocytes specific for* T. cruzi* in a wide variety of patients with Chagas disease without having to perform HLA typing. Furthermore, it was noted by the authors that the frequency of specific CD8^+^ T lymphocytes producing IL-2 was significantly reduced in chronically infected patients [36]. Considering that the parasite load is extremely low in these individuals, the authors proposed that long-term parasite persistence can result in a specific T lymphocyte population with low capacity for self-renewal. While this work was only focused on the response to TS proteins, thus only examining a fraction of all the possible CD8^+^ T lymphocytes specific for* T. cruzi*, it is significant that more than 50% of the evaluated subjects responded to at least one TS epitope.

### 5.2. Experimental Murine Models

A few hours after penetrating the host cell,* T. cruzi* escapes from the parasitophorous vacuole and enters the cytosol, where it differentiates into amastigotes and begins its replication. Once the parasite is in the cytosol, its secreted antigens become available to be processed and presented via MHC-I [69]. The relevance of CD8^+^ T lymphocytes in immunity against experimental infection with* T. cruzi* has been demonstrated by different methods. Studies have used *β*2-microglobulin-deficient mice, which lack cells restricted to MHC-I molecules (such as NK cells and CD8^+^ T lymphocytes), mice deficient in the CD8 molecule, or mice depleted of CD8^+^ T cells using monoclonal antibodies. In all cases, the absence of CD8^+^ T cells leads to extreme susceptibility to experimental infection [5]. Specific CD8^+^ T cells promote resistance to infection by* T. cruzi *through IFN-*γ*, but direct perforin-mediated cytotoxic activity can also play an important role depending on the strain of* T. cruzi* [5, 27, 28].

The mechanisms involved in the processing and presentation of these antigens to CD8^+^ T cells are unknown. There is evidence that CD11c^+^ dendritic cells are important for the initiation of the immune response that occurs in the draining lymph nodes [65, 70]. Afterwards, the CD8^+^ T lymphocytes recirculate and enter the spleen. This requires the expression of the chemotactic receptor S1PR1 (sphingosine 1 phosphate receptor) on the surface of lymphocytes, as egress can be blocked by use of the drug FTY720 [65].

The response of CD8^+^ T lymphocytes during experimental infection with* T. cruzi* has kinetics distinct from those observed with viruses or bacteria that cause self-limiting infections, such as influenza, LCMV, or* Listeria.* In these cases, the peak of the immune response measured by the frequency of specific CD8^+^ T lymphocytes is 7 to 15 days [71]. After* T. cruzi* infection, we and others have observed that the peak of the immune response occurs after approximately 30 days [27, 39, 72, 73]. These delayed kinetics of the immune response most likely allow the parasite to establish tissue infection before they can be eliminated by CD8^+^ T lymphocytes. The precise mechanisms that control this altered kinetics are not known, but we have observed that the parasite load is an important factor controlling the timing of the immune response. Thus, increasing the parasite inoculum leads to acceleration of the parasitemia/infection onset, which in turn triggers acceleration of specific CD8^+^ T lymphocyte generation [73].

**Figure 1:**
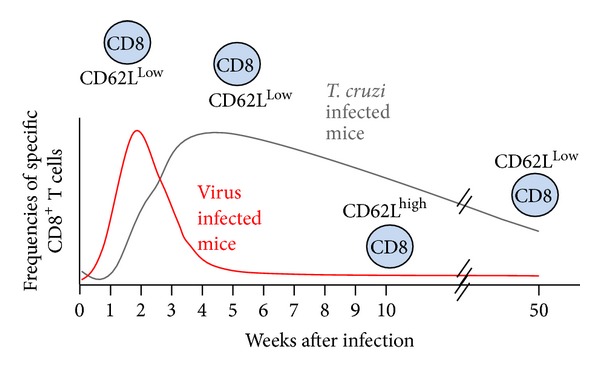
Kinetics of the immune response mediated by CD8^+^ lymphocytes after experimental infection with viruses or with* T. cruzi*.

In contrast to what is seen in common viral or bacterial infections, we do not observe a marked decrease (contraction) in the immune response. As shown in Figure 1, the frequencies of specific CD8^+^ T cells remain high for long periods of time. Furthermore, the phenotype of these CD8^+^ T lymphocytes is maintained as T effector memory (TEM) cells through this period, and this is linked to parasite persistence. Effective treatment with an antitrypanocidal drug led to the contraction of the number of CD8^+^ T cells and a change in their phenotype to CD62L^Low^ [74].

**Figure 2:**
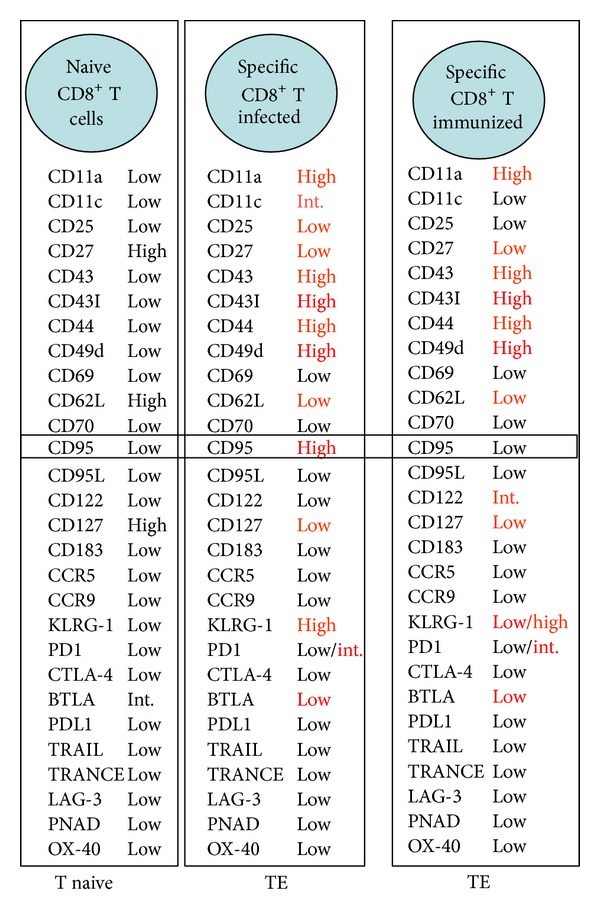
Surface molecules expressed by specific CD8^+^ T lymphocytes following infection with* T. cruzi* and vaccination with AdASP-2.

Importantly, the immune response mediated by CD8^+^ T cells generated after experimental infection does not always allow host survival. Mice from susceptible strains, such A/Sn, uniformly die after infection with few parasites [75]. However, after infection of resistant mice, such as C57BL/6, the immune response mediated by CD8^+^ T lymphocytes protects the animals from death. Nevertheless, these animals are unable to completely eliminate the parasites, which drives them to the chronic phase of infection. Thus, under no circumstances can the immune response mediated by CD8^+^ T lymphocytes eliminate* T. cruzi*. In contrast, genetic vaccination with a DNA plasmid and recombinant adenovirus expressing an immunodominant* T. cruzi* antigen amastigote surface protein 2 (ASP-2) is able to induce a potent immune response mediated by CD8^+^ T lymphocytes that protects highly susceptible A/Sn mice [28, 76]. These animals not only survive the acute infection but also do not develop chronic Chagas disease and have no detectable circulating parasites. This observation has created a paradox, since both experimental infections and genetic immunizations induce CD8^+^ T cells that are capable of secreting the antiparasitic mediator IFN-*γ* and mediating potent cytotoxic lymphocyte activity* in vivo*. To explain this paradox, we hypothesize that the specific CD8^+^ T lymphocytes induced by the adenoviral vector expressing the ASP-2 antigen (AdASP-2) or by experimental infection present distinct properties that either confer immunoprotective ability in the former or prevent an effective response in the latter. In order to identify this factor, we made a detailed comparison of the two immune responses. We observed that there was a high degree of similarity in the immune responses induced by AdASP-2 and by infection with* T. cruzi*. After 19 days, the immune responses had similar magnitudes and were mediated by multifunctional lymphocytes capable of translocating the CD107a molecule to the cell surface as well as accumulating IFN-*γ* and TNF-*α* after* in vitro* stimulation with cognate peptide [75]. Moreover, these specific lymphocytes were highly cytotoxic* in vivo*. Among the few differences, there was a markedly accelerated expansion of the CD8^+^ T cell immune response induced by AdASP-2 compared to* T. cruzi* infection [75]. When we evaluated the proliferation of specific CD8^+^ T lymphocytes, we found that a higher frequency of cells proliferated following infection when compared to immunization with AdASP-2 [75]. However, as mentioned above, their frequencies were similar. This discrepancy was explained by a significant increase in the frequency of specific CD8^+^ T lymphocytes with a proapoptotic phenotype in infected animals compared to immunized animals. Therefore, the main difference observed when comparing the two immune responses was the viability (fitness) of specific CD8^+^ T lymphocytes. Whereas immunization with AdASP-2 induced highly viable specific CD8^+^ T cells, infection led to intense expansion and apoptosis. In order to determine the molecular basis for this phenomenon, we analyzed a total of 28 surface molecules, including adhesion molecules, functional markers, and receptors involved in T lymphocyte survival (Figure 2). The main difference observed was that specific CD8^+^ T cells induced by experimental infection showed a higher expression of apoptotic CD95 (Fas) receptor compared with specific CD8^+^ lymphocytes from mice vaccinated with AdASP-2 [75]. Not only these cells expressed high levels of CD95 on the surface, but signaling through this molecule* in vitro* with anti-CD95 mAb led to their death. In contrast, specific CD8^+^ T lymphocytes induced by vaccination with AdASP-2 were refractory to death induced by anti-CD95 [75].

**Figure 3:**
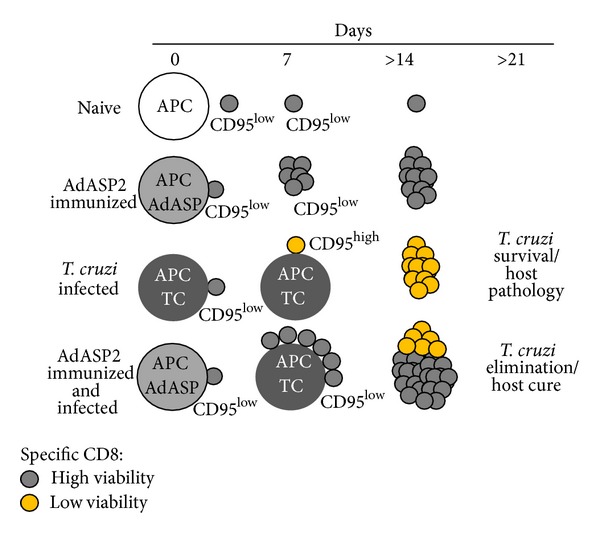
Proposed model for activation of specific CD8^+^ lymphocytes during infection of* T. cruzi* in naive or AdASP-2 immunized mice. Specific CD8^+^ T cells activated with APC containing* T. cruzi* antigen induce higher levels of expression of CD95 and low viability of T cells. This poor viability leads to a suboptimal response and host pathology (death or chronic infection). In contrast, the contact of APC pulsed with recombinant ADASP-2 activates specific CD8^+^ T cells with low expression of CD95. In the second contact with APC pulsed with* T. cruzi* antigen, specific CD8^+^ T cells with CD95^low^ phenotype develop a strong immune response driving parasite elimination and promoting cure of the infection.

These striking differences in the quality of the immune response induced by immunization with the AdASP-2 and by* T. cruzi* infection provide an important basis to explain the suboptimal immune response mediated by CD8^+^ T cells during infection. In addition, this difference may also explain the success of genetic vaccination with recombinant adenoviral vectors. Indeed, concomitant injection of AdASP-2 and* T. cruzi* led to (i) a strong specific immune response mediated by CD8^+^ T lymphocytes; (ii) lower expression of CD95, which led to resistance to anti-CD95-mediated apoptosis; (iii) protective immunity against experimental infection with* T. cruzi* [77] (Figure 3).

Another important factor that characterizes the immune response mediated by CD8^+^ T lymphocytes during experimental* T. cruzi* infection in mice is the fact that it is restricted to few epitopes. This occurs despite the great number of possible CD8 epitopes present in the parasite. This phenomenon is called immunodominance. All the immunodominant epitopes described thus far are expressed by members of the TS family of* T. cruzi*.

Several molecular mechanisms leading to the immunodominance of CD8^+^ T cells during infection are known, some of which are related to the formation of the peptide-MHC-I complex on the surface of APC. The following are important for the formation of these complexes: (i) abundance of the antigen; (ii) availability of APC; (iii) enzymatic processing by proteasomes and other proteolytic enzymes; (iv) translocation of peptides into the endoplasmic reticulum by transporter proteins; (v) binding of peptides to MHC-I. The second group of factors that influence immunodominance are related to the activation of CD8^+^ T lymphocytes: (i) frequency of cell precursors in the repertoire capable of recognizing the peptide-MHC-I complex; (ii) affinity of the T cell receptors; (iii) CD8^+^ T lymphocyte competition by the APC during priming. Other factors, such as regulation of specific immune responses, the ability of pathogens to mutate their recognized epitopes, expression of specific antigens on the different evolutionary stages, modulation of antigen expression at different stages of infection, and direct inhibition of mechanisms involved in the formation of the peptide-MHC-I complex could also theoretically influence immunodominance, but examples have not yet been described.

Due to the genetic restriction by MHC-I molecules in the case of CD8^+^ T cells, 90% of immunodominance is linked to the fact that only 1% of peptides can bind MHC-I with affinity sufficient to induce a response mediated by CD8^+^ T lymphocytes. This is related to the structure of MHC-I rather than the selective pressure by an organism to prevent the host CD8^+^ T lymphocyte response [78]. Because of the genomic complexity of many infectious agents, most studies seeking to define the molecular basis of immunodominance of CD8^+^ T cell-mediated immunity have used viral models. In a review article on immunodominance [79], they illustrate the evolution of immune restriction mediated by CD8^+^ lymphocytes during vaccinia virus infection. The vaccinia genome has the capacity to generate approximately 175,000 peptides of 8, 9, or 10 mer (without considering the possibility of alternative sequences of reads and posttranslational modifications). However, only 20% of these peptides are released after processing by the proteasome and other proteases (35,000 peptides). Although transport to the ER via TAP influences the selection of peptides that are made available to bind MHC-I, such transport has little effect on the restriction of epitopes during vaccinia infection, and approximately 30,000 peptides are transported. Even so, approximately 1% of these peptides bind MHC-I (150 peptides). Once the peptide-MHC-I complex is exposed on the surface of the APC, the repertoire of CD8^+^ T lymphocytes can recognize at least 50% of these epitopes (75 peptides). However, only 50 peptides are recognized, and these make up 90% of the response to vaccinia virus. Finally these 50 vaccinia peptides that are recognized during experimental infection, eight represent more than 50% of the CD8^+^ T lymphocyte response observed against infection.

As mentioned above, the antigen targets of the immune response mediated by CD8^+^ T cells during* T. cruzi* experimental infection are of TS family proteins [27, 72, 73, 80, 81] (Table 1). The molecular basis and biological significance of immunodominance are unknown. It is possible that the restriction of the number of known parasite epitopes may be important for establishing the necessary balance between the parasite and the host in order to keep the host alive without eliminating the pathogen, thus leading to chronic infection [5, 82]. Immunodominant epitopes vary by* T. cruzi* strain, such that an epitope can be immunodominant during infection with one strain and subdominant with another in genetically identical hosts [39, 72].

Despite evidence of strong immunodominance during* T. cruzi *experimental infection, to date only one study has attempted to determine the cellular/molecular basis for this phenomenon. In this study, it was reported that infected homozygous C57BL/6 mice or heterozygous F1 mice (C57BL/6 X BALB/c) had similar CD8^+^ T lymphocytes responses to the MHC-I H-2K^b^-restricted epitope VNHRFTLV. In contrast, the immune response to the MHC-I H-2K^d^-restricted epitope IYNVGQVSI was significantly reduced in heterozygous animals when compared to homozygous BALB/c mice [72]. Such a discrepancy could not be attributed to the CD8^+^ T lymphocyte repertoire or to low MHC-I H-2K^d^ expression on the APC surface of heterozygous mice. This phenomenon, which restricted the immune response and generated strong immunodominance, could be reproduced in other heterozygote strains of mice after infection with* T. cruzi*. However, there was no difference in the immune responses of homozygote and heterozygote animals following immunization with the AdASP-2 expressing* T. cruzi *antigen [72]. Finally, in the same study, C57BL/6 mice infected with two different strains of* T. cruzi,* both the Y and CL-Brener strains, responded to the two immunodominant epitopes simultaneously. Based on these results, it was proposed that the strong immunodominance induced during infection with* T. cruzi* occurred by the competition of CD8^+^ T lymphocytes generated during priming for a limited number of infected APCs. This competition for APC is also called competitive priming or immunodomination and has been reported in some cases of experimental infection [72, 83].

These observations were independently confirmed in studies showing that CD8^+^ T cells of H-2^a^ infected mice recognized a single immunodominant epitope in the ASP-2 antigen. In contrast, CD8^+^ T cells from mice immunized with recombinant genetic vaccines expressing the same* T. cruzi* antigen recognized two other subdominant epitopes in addition to the immunodominant epitope [84].

Despite the immunoprotective role of CD8^+^ T lymphocytes during the acute phase of experimental* T. cruzi* infection, recent studies have proposed that a subpopulation of these lymphocytes is eventually responsible for further damage to heart tissue in the chronic phase. These lymphocytes produce perforin, but not IFN-*γ*, and have a greater propensity to migrate to the heart tissue, causing tissue damage leading to CCC [85].

### 5.3. CD8^+^ T Cell Mediated Immunity for Vaccine Development

Based on the proven antiparasitic activity of CD8^+^ T cells, several groups have tried to elicit specific CD8^+^ T cells for vaccine development against Chagas disease. For that purpose, subunit vaccines based on plasmid DNA, viral, and bacterial vectors were used. The antigens pursued for recombinant vaccines include* T. cruzi trans*-sialidase, cruzain (a cysteine proteinase), and the amastigote surface proteins 2, 3, 4, TcG1, TcG2, TcG4, TSA-1, and Tc24 (review references [23, 80, 86–90]). In addition to subunit vaccines, other groups are using attenuated parasites [90]. A more detailed list of the vaccination studies performed with subunit vaccines is shown in Table 2. Overall, the results of mouse vaccinations provide strong evidence that the induction of specific CD8^+^ T cells can elicit protective immunity against acute phase symptoms estimated by parasitemia and survival. In some cases, vaccination also reduced or eliminated chronic symptoms in these mouse models.

An important question is whether vaccination can achieve complete parasite elimination from the host. In most protocols of experimental vaccination, residual parasites are still present and are mostly within tissues rather than circulating in the blood. Therefore, vaccination may at least lead to a decrease or cessation in transmission. Vaccinated mice rarely show symptoms of acute or chronic disease. Therefore, even if they carry tissue parasites they will be asymptomatic. This picture resembles the ~60% of chagasic patients who also maintain residual parasites for their entire lives yet still remain asymptomatic (indeterminate form of the disease). Thus, it would be ethical to propose that a satisfactory vaccination goal could be to lower the frequencies of symptomatic individuals even if that means increasing the frequency of asymptomatic individuals.

During our recent studies of genetic immunization, we used the heterologous prime-boost vaccination regimen consisting of priming with plasmid DNA followed by a booster injection with replication defective human adenovirus type 5, both carrying the ASP-2 gene of* T. cruzi*. In these studies, we observed that our immunization protocol induced TE cells (CD11a^High^, CD44^High^, CD127^Low^, and CD62L^Low^). These cells formed a stable pool of functional long-lived CD8^+^ TEM cells (CD11a^High^, CD44^High^, CD127^High^, and CD62L^Low^). Their long-term survival required a functional IL-12/IL-23 signaling pathway. Most relevant for vaccine development was the fact that these cells were resistant to immunological erosion and mediated strong protective immunity against experimental systemic infection with* T. cruzi* [76].

According to the current immunological paradigm of vaccines, protective immunity is mainly mediated by T central memory (TCM) cells, which proliferate intensely during recall responses. After an infectious challenge, specific TCM cells differentiate to TE cells, proliferate, and eliminate the pathogen. This concept established that, after vaccination, TCM cells act similarly to self-curing viral infections, such as acute LCMV [91].

Based on this paradigm, we evaluated the importance of CD8^+^ T cell differentiation, proliferation, and recirculation to mediate their antiparasitic effects. In our case, we only had a TEM pool to stimulate. We observed that specific TEM cells differentiated into cells with a KLRG1^High^ CD27^Low^ CD43^Low^ CD183^Low^ *T-bet* ^High^ *Eomes* ^Low^ phenotype and were capable of simultaneously producing the antiparasitic mediators IFN-*γ* and TNF-*α*. Subsequently, we observed that the specific CD8^+^ T cells did not participate in a strong anamnestic immune response, and the protective immunity they mediated was insensitive to treatment with the cytostatic toxic agent hydroxyurea, which drastically reduced their proliferation [92].

We also evaluated the importance of T cell recirculation by administering the immunosuppressive drug FTY720 to vaccinated mice after challenge with* T. cruzi*. This drug reduces lymphocyte recirculation by interfering with T cell signaling via sphingosine-1-phosphate receptor-1 (S1Pr1). This interference results in sustained inhibition of S1Pr1 signaling, effectively trapping T cells within the secondary lymphoid without inhibiting T cell activation. FTY720 administration significantly impaired protective immunity, supporting the hypothesis that T cell recirculation is critical for protective immunity [65].

**Figure 4:**
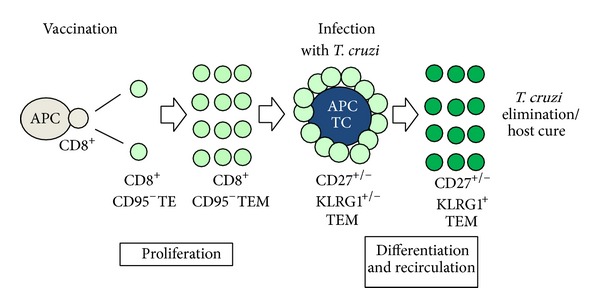
Proposed model for activation of specific CD8^+^ lymphocytes expanded by genetic vaccination after infection with* T. cruzi*.

Based on these observations, we propose a model whereby large amounts of antigen-experienced CD8^+^ TEM cells present following heterologous prime-boost vaccination that differentiate and recirculate, rather than proliferate, are critical for protective immunity Figure 4. Recent studies using the murine LCMV infection model reported that CD8^+^ T cells that provide long-term protective immunity are a subpopulation of TEM cells with the same phenotype as our T cells (CD44^High^ CD62^Low^ KLRG1^High^ CD27^Low^ CD43 (1B1)^Low^ CD183^Low^ T-bet^High^ *Eomes* ^Low^) and not classical TCM. These cells have limited proliferative ability, recirculate, and are also critical in the resistance to infection with* Listeria monocytogenes* and vaccinia [93]. Together, these observations point to a novel CD8^+^ T cell population that provides long-term memory and can be used for T cell vaccine development. Additionally, these two observations challenge the paradigm that memory T lymphocytes need to proliferate in order to mediate protective immunity and suggest that recirculation is indeed fundamental. This idea had not been properly explored and may have important implications in the development of new vaccines.

A current limitation of the experimental model of* T. cruzi* infection is the absence of information about the precise location where CD8^+^ T lymphocytes eliminate infected cells. Because* T. cruzi* can infect many cell types and cause systemic (or not) infection, it will be important to determine where CD8^+^ T lymphocytes encounter infected cells. The fact that our vaccinated animals were resistant to challenge with* T. cruzi* infection by different routes (i.p. and s.c.) suggests that it may also be useful for natural infections.

## 6. Concluding Remarks

Studies on the role of CD8^+^ T cells during* T. cruzi* infection have elucidated extremely important information regarding the interface of host/*T. cruzi* interactions and the equilibrium they achieve to establish chronic infection. In addition, they have opened new avenues for the development of a still elusive veterinarian or human vaccine against Chagas disease.

## Figures and Tables

**Table 1:** Immunodominant epitopes recognized by murine CD8^+^ lymphocytes after infection with *T. cruzi*.

Antigen	Epitope	MHC	*T. cruzi *strain	Reference
TS	IYNVGQVSI	H-2K^d^	Y	[37]
ASP-2	VNHRFTLV	H-2K^b^	Y	[27]
ASP-2	TEWETGQI	H-2K^k^	Y	[38]
TSA	ANYDFTLV	H-2K^b^	Brazil	[39]
TSA	ANYKFTLV	H-2K^b^	Brazil	[39]

**Table 2:** Summary of the different antigen delivery systems used for the induction of CD8^+^ T cells against experimental *T. cruzi* infection.

Delivery system	Adjuvant	*T. cruzi* antigen/gene	Reference
Rec. protein	Freund's adjuvant	TS	[40]
Rec. Protein	CpG ODN	TS	[41]
Rec. Protein	CpG ODN	Cruzipain	[42]
Rec. protein	Alum/CpG ODN	ASP-2	[38]
			[43]
Plasmid DNA	None	TSA-1	[44]
			[45]
			[46]
Plasmid DNA	IL-12	TSA	[47]
			[48]
Plasmid DNA	None	TS	[49]
			[50]
			[41]
Plasmid DNA	None	ASP-1	[51]
Plasmid DNA	None	ASP-2	[51]
			[52]
			[53]
			[54]
			[55]
Plasmid DNA	None	ASP-3	[56]
Plasmid DNA	None	ASP-4	[56]
Plasmid DNA	None	CRP	[57]
Plasmid DNA	None	KMP11	[58]
Plasmid DNA	None	Tc24	[45]
Plasmid DNA	IL-12/GM-CSF	TcG1	[59]
Plasmid DNA	IL-12/GM-CSF	TcG2	[59]
Plasmid DNA	IL-12/GM-CSF	TcG4	[59]
Rec. adenovirus	None	TS	[60]
		ASP-2	[60]
Rec. *Salmonella *	None	Cruzipain	[61]
Sendai virus	None	ASP-2	[62]
Yellow fever 17D	None	ASP-2	[63]
Rec. adenovirus + Rec. MVA	None	TSA	[64]
Plasmid DNA + Rec. Adenovirus	None	ASP-2	[28]
Plasmid DNA + Rec. Adenovirus	None	ASP-2	[65]
Rec. influenza + Rec. Adenovirus	None	ASP-2	[65, 66]
Plasmid + Rec. MVA	IL-12/GM-CSF	TcG2/TcG4	[67]
Plasmid + Rec. protein	IL-12/GM-CSF	TcG1/TcG2/TcG4	[68]
